# Adding Preoperative Oral Antibiotics to Mechanical Bowel Preparation Reduces Surgical Site Infections in Elective Colorectal Surgery: A Meta-Analysis of Randomized Controlled Trials

**DOI:** 10.3390/medicina62061161

**Published:** 2026-06-15

**Authors:** Héctor Guadalajara, Alicia Putan, Mariano García Arranz, Miguel León-Arellano, Raquel Sanz-Baro, Jose Manuel Ramirez, Damián García-Olmo

**Affiliations:** 1 Department of Surgery, School of Medicine, Universidad Autónoma de Madrid, 28029 Madrid, Spain; 2 Department of General and Digestive Surgery, Hospital Universitario Fundación Jiménez Díaz, Av. Reyes Católicos 2, 28040 Madrid, Spain; 3 Department of Gynecology and Obstetrics, Hospital Universitario Fundación Jiménez Díaz, Av. Reyes Católicos 2, 28040 Madrid, Spain; 4 Department of Surgery, School of Medicine, University of Zaragoza, 50009 Zaragoza, Spain; 5 Department of Surgery, Aragon Institute for Health Research (IIS Aragon), 50009 Zaragoza, Spain

**Keywords:** cathartics, anti-bacterial agents, antibiotic prophylaxis, colorectal surgical procedures, surgical wound infection, randomized controlled trials as topic, systematic review, meta-analysis

## Abstract

*Background and Objectives*: Surgical site infections (SSIs) remain common after elective colorectal surgery. This systematic review and meta-analysis evaluated whether adding oral antibiotic bowel preparation (OAB) to mechanical bowel preparation (MBP) reduces SSIs compared with MBP alone. *Materials and Methods*: PubMed, the Cochrane Library, Scopus, and ClinicalTrials.gov were searched for English-language randomized controlled trials published from January 2005 to January 2025. Eligible trials enrolled adults undergoing elective colorectal surgery and compared MBP+OAB versus MBP alone, with standard intravenous prophylaxis in both groups. The primary outcome was overall SSI; secondary outcomes were incisional SSI and organ-space SSI. Risk of bias was assessed with RoB 2, certainty with GRADE, and odds ratios (ORs) were pooled using DerSimonian–Laird random-effects models. The protocol was prespecified but not prospectively registered. *Results*: Twelve trials including 4073 patients were included (MBP+OAB, n = 2069; MBP, n = 2004). MBP+OAB reduced overall SSI (OR 0.53, 95% CI 0.37–0.75; *p* < 0.001; I^2^ = 62.5%; 95% prediction interval 0.17–1.66), incisional SSI (OR 0.52, 95% CI 0.34–0.80; *p* = 0.003; I^2^ = 57.5%), and organ-space SSI (OR 0.63, 95% CI 0.45–0.88; *p* = 0.007; I^2^ = 8.3%). The effect was preserved in metronidazole-containing regimens (OR 0.46, 95% CI 0.33–0.65), but this subgroup was exploratory. Excluding high-risk-of-bias studies supported the primary result. Publication-bias assessment was underpowered. Overall and organ-space SSI were moderate-certainty outcomes; incisional SSI was low-certainty, and anastomotic leak was very low-certainty. *Conclusions*: In contemporary elective colorectal surgery when MBP is used, adding preoperative OAB probably reduces SSIs. Findings do not establish whether OAB alone is sufficient or whether MBP is necessary; stewardship-relevant outcomes remain insufficiently reported. Funding was provided by ISCIII grant PI25/01285.

## 1. Introduction

Colorectal surgery carries a high risk of postoperative complications due to the dense bacterial colonization of the large intestine. Surgical site infections (SSIs) occur in up to approximately one-third of patients undergoing colorectal resections [1]. These infections, together with related complications such as anastomotic leak, represent a major clinical challenge, as they increase patient morbidity, prolong hospital stay, and escalate healthcare costs. In addition, SSIs and other postoperative complications may adversely affect long-term oncological outcomes, including increased risks of local recurrence and distant metastasis in colorectal cancer [2]. Reducing postoperative infection rates is therefore critical to improving both short- and long-term patient outcomes.

Preoperative bowel preparation has long been explored as a strategy to mitigate these risks. Historically, mechanical bowel preparation (MBP), consisting of cathartic cleansing of the colon, was considered standard practice. Early experimental work suggested that effective mechanical cleansing could substantially reduce fecal load and bacterial burden within the colon. MBP was also believed to facilitate surgical handling of the bowel and construction of a safe anastomosis. However, evidence accumulated over the past two decades has challenged the clinical benefit of MBP when used alone. Multiple randomized trials and systematic reviews have demonstrated that MBP by itself does not significantly reduce SSI rates [3,4,5,6]. Some studies have even suggested that MBP may increase postoperative complications, including ileus and anastomotic leakage. A Cochrane review reported no reduction in SSI with MBP alone and highlighted potential harms [4], while a comparative effectiveness study found MBP to be associated with higher complication rates, calling into question its routine use in colorectal surgery [7]. Consequently, by the early 2010s, many surgeons had abandoned routine MBP in elective colorectal procedures.

Importantly, most early trials comparing MBP with no bowel preparation did not incorporate an oral antibiotic component in the preoperative regimen. As early as the 1970s, studies demonstrated that the addition of non-absorbable oral antibiotics (OAB) to MBP could further reduce SSI rates, prompting renewed interest in oral antibiotic bowel preparation. Nevertheless, adoption of this strategy has been inconsistent across institutions and regions. More recent randomized trials have supported a role for oral antibiotics in colorectal surgery, although the accompanying mechanical preparation strategy has varied across studies [8,9]. These findings have revived the debate regarding whether prophylactic antibiotics should be administered in addition to MBP and through which route—intravenous (IV), oral, or both. In contemporary practice, IV antibiotic prophylaxis at induction of anesthesia is universal; however, the incremental benefit of adding oral antibiotics remains a subject of controversy.

Multiple randomized trials and meta-analyses have addressed this question with heterogeneous results. Several studies have reported that combining oral and systemic antibiotics significantly reduces SSI rates compared with systemic antibiotics alone [10,11]. Conversely, randomized evidence has not uniformly demonstrated a significant advantage of adding oral antibiotics to standard IV prophylaxis. These conflicting findings may reflect heterogeneity in patient populations, surgical procedures, antibiotic regimens, and definitions of SSI across studies. Recent high-level evidence, including a Cochrane review, a network meta-analysis, and additional trial-level meta-analyses, has attempted to clarify these discrepancies and generally supports the addition of oral antibiotics to MBP in reducing postoperative infectious complications [12,13,14]. Nevertheless, variability in study quality and protocols continues to generate uncertainty.

When oral antibiotics are used for bowel preparation, adequate coverage of both aerobic and anaerobic bacteria implicated in colorectal SSIs is essential. The colonic microbiota comprises a complex mixture of aerobic and anaerobic organisms, and regimens targeting only one component are likely to be less effective. Accordingly, most oral antibiotic protocols combine a non-absorbable aminoglycoside, such as neomycin or kanamycin, to provide aerobic Gram-negative coverage, with metronidazole to target anaerobic bacteria. Ensuring sufficiently high intraluminal antibiotic concentrations at the time of surgery is also critical. While the classic Nichols regimen combined neomycin with erythromycin, limitations in drug availability and evolving resistance patterns have led to the adoption of alternative regimens, including fluoroquinolones administered alongside metronidazole.

Metronidazole warrants particular attention in this context. In addition to its potent antimicrobial activity against anaerobes, emerging experimental evidence suggests that metronidazole may inhibit the expression of collagen-degrading enzymes, such as matrix metalloproteinases, which are involved in tissue remodeling and wound healing [15]. This raises the hypothesis that metronidazole-containing regimens may not only reduce infectious complications but also positively influence anastomotic integrity and wound healing. In light of these considerations, our meta-analysis was designed to include a prespecified subgroup analysis focusing on regimens incorporating metronidazole.

In summary, while mechanical bowel preparation alone has not demonstrated consistent benefit in reducing postoperative infections, the role of oral antibiotic bowel preparation remains an area of active debate. High-quality evidence is required to clarify the efficacy of combining MBP with oral antibiotics in contemporary colorectal surgery. We therefore conducted a systematic review and meta-analysis of randomized controlled trials to evaluate whether MBP combined with oral antibiotics is superior to MBP alone in preventing surgical site infections in elective colorectal surgery. Secondary infection-related outcomes and prespecified exploratory subgroup analyses, including metronidazole-containing regimens, were also examined to inform clinical practice and support protocol standardization.

## 2. Materials and Methods

### 2.1. Study Design and Protocol

This systematic review and meta-analysis was conducted and reported in accordance with the Preferred Reporting Items for Systematic Reviews and Meta-Analyses (PRISMA) 2020 guidelines [16]. A completed PRISMA 2020 checklist is provided as Supplementary Table S1. The methodology was prespecified in a study protocol, but the review was not prospectively registered in PROSPERO or another public registry. The research question was defined according to the PICO framework: population—adult patients undergoing elective colorectal surgery; intervention—mechanical bowel preparation plus oral antibiotics (MBP+OAB); comparison—mechanical bowel preparation alone (MBP); outcome—postoperative surgical site infection (SSI). Both incisional (superficial or deep) and organ-space SSIs were considered.

The present analysis focused on the additive effect of preoperative oral antibiotics, as standard intravenous (IV) antibiotic prophylaxis at anesthetic induction was administered in all included trials.

### 2.2. Literature Search Strategy

A comprehensive literature search was performed to identify randomized controlled trials (RCTs) evaluating the effect of oral antibiotic bowel preparation in colorectal surgery. We searched PubMed, the Cochrane Library, and Scopus for studies published between January 2005 and January 2025, restricted to the English language. ClinicalTrials.gov (Bethesda, MA, USA) was also consulted as a trial registry. The final search for all databases and ClinicalTrials.gov was completed in January 2025.

The restriction to studies published from 2005 onward was defined a priori to focus on contemporary colorectal surgery, given the major changes in perioperative care, surgical technique, infection-prevention strategies, and enhanced recovery pathways that have occurred since the mid-2000s [17,18].

Search strategies combined controlled vocabulary and free-text terms related to colorectal surgery, bowel preparation, oral antibiotics, surgical site infection, and randomized trials. Equivalent strategies were adapted for each database. The full reproducible search strategies for PubMed, the Cochrane Library, and Scopus, including Boolean operators, date limits, language restrictions, and records retrieved, are provided in Supplementary Table S2. ClinicalTrials.gov was used as a registry source to screen for potentially relevant registered or completed trials. Reference lists of eligible studies and relevant systematic reviews were manually screened. Broader grey literature sources, such as conference abstracts, dissertations, preprints, and unpublished reports, were not systematically searched because this review was designed to synthesize full-text published randomized trials with sufficient methodological detail and extractable outcome data.

### 2.3. Eligibility Criteria

Studies were included if they met the following criteria: (1) randomized controlled trial design; (2) adult patients (≥18 years) undergoing elective colorectal surgery; (3) intervention consisting of preoperative mechanical bowel preparation plus oral antibiotics; (4) comparison group receiving mechanical bowel preparation without oral antibiotics; and (5) reporting postoperative SSI outcomes within the surveillance period defined by the original trial authors, provided that the outcome definition was compatible with standard SSI surveillance criteria. Follow-up duration was recorded and considered in the interpretation of heterogeneity and risk of bias.

Only randomized controlled trials were included. This criterion was prespecified because the objective of the review was to estimate the comparative efficacy of adding oral antibiotic bowel preparation to mechanical bowel preparation while minimizing confounding by indication, selection bias, and baseline differences between intervention groups. Observational studies were excluded because the decision to administer oral antibiotics may be influenced by institutional protocols, surgeon preference, patient risk profile, type of colorectal procedure, ERAS implementation, and other perioperative factors that may not be fully captured or adjusted for in non-randomized designs [19,20].

Trials were eligible regardless of the specific oral antibiotic agent or absorbability profile, dose, timing, or combination regimen, provided that oral antibiotics were administered preoperatively. Both single-agent and combination protocols were accepted. To ensure comparability, all patients were required to have received standard IV antibiotic prophylaxis. Pediatric and emergency surgery studies were excluded. When multiple publications reported overlapping cohorts, the most comprehensive report was included.

### 2.4. Study Selection

**Figure 1:**
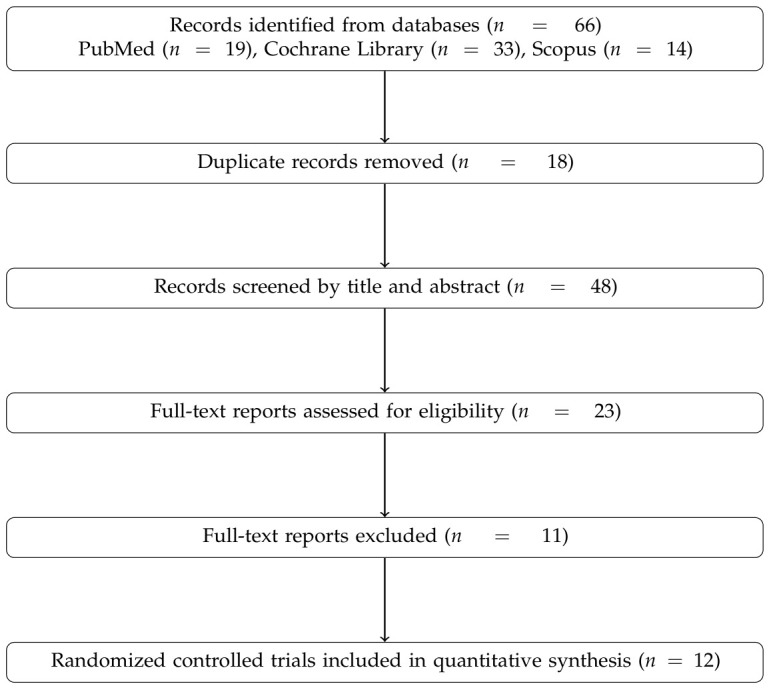
PRISMA 2020 flow diagram of study selection.

Records were merged and duplicates removed. Two reviewers independently screened titles and abstracts. Full texts were reviewed against inclusion criteria, with disagreements resolved by consensus. The selection process is summarized in the PRISMA flow diagram (Figure 1).

### 2.5. Data Extraction

Data were extracted using a predefined form, including study characteristics, patient numbers, bowel preparation protocols, outcome definitions, SSI incidence, follow-up duration, and adverse events. Derived data were calculated when necessary. All data were cross-checked by two reviewers.

### 2.6. Risk-of-Bias Assessment

Risk of bias was assessed using the Cochrane Risk of Bias 2.0 (RoB 2) tool [21]. Domains included randomization, deviations from intended interventions, missing data, outcome measurement, and selective reporting. Overall trial judgments were also used in an additional sensitivity analysis excluding studies at high overall risk of bias for the primary outcome.

### 2.7. Outcomes

SSI was defined according to the criteria used by the original trial authors, provided that these were compatible with standard CDC or equivalent surveillance definitions [22]. In general, SSI was considered an infection related to the surgical procedure occurring during the postoperative surveillance period and classified as incisional (superficial or deep) or organ-space infection. A superficial infection was limited to the skin and subcutaneous tissue of the incision, whereas deep incisional SSI involved deeper incisional tissues such as fascia or muscle. Organ/space SSI involved any anatomical site opened or manipulated during the operation, deeper than the fascia or muscle, including intra-abdominal or pelvic infection.

The primary outcome was overall SSI, defined as the occurrence of any incisional (superficial or deep) or organ-space SSI during the follow-up period reported by each study. Secondary outcomes were incisional SSI and organ-space SSI when arm-level data were available. Incisional SSI was harmonized using the closest trial-reported incisional category; when superficial and deep incisional SSI were reported separately, both were summed. When individual studies used study-specific wording or surveillance rules, SSI data were extracted according to the definitions reported in each trial, provided that they were compatible with standard CDC or equivalent criteria. Deep incisional SSI contributed to the harmonized incisional endpoint when reported separately, but it was not meta-analyzed as a separate independent endpoint because it was not consistently reported across trials. Anastomotic leak was considered a clinically relevant exploratory outcome. However, because leak definitions, denominators, and reporting formats were inconsistent across trials, it was synthesized descriptively rather than meta-analyzed.

### 2.8. Statistical Analysis

All analyses were performed in Python 3.11. Data handling was performed with pandas and numpy, statistical calculations used SciPy where applicable, graphical displays were generated with matplotlib, and meta-analyses were conducted with the statsmodels.stats.meta_analysis module. For each analyzed outcome, study-specific 2×2 tables were reconstructed from the numbers of events and non-events in the intervention and control groups. For multi-arm trials relevant to this review question, oral-antibiotic arms were combined into a single MBP+OAB group by summing events and sample sizes before reconstruction of the 2×2 table, so that each randomized participant contributed only once to the pairwise comparison with MBP alone [23].

Let *a* denote the number of events in the MBP+OAB group, *b* the number of non-events in the MBP+OAB group, *c* the number of events in the MBP group, and *d* the number of non-events in the MBP group. The study-specific effect measure was the odds ratio (OR), calculated as

$$OR=\frac{a/b}{c/d}=\frac{ad}{bc}.$$

For meta-analysis, odds ratios were transformed to the natural logarithmic scale,

y=log(OR),

and the within-study variance and standard error were calculated as

$$\mathrm{Var}\left(y\right)=\frac{1}{a}+\frac{1}{b}+\frac{1}{c}+\frac{1}{d},\;SE\left(y\right)=\sqrt{\mathrm{Var}(y)}.$$

If any cell in a study-specific 2×2 table contained zero events, a continuity correction of 0.5 was added to all four cells of that table before calculation of the OR and its variance [23]. Studies with heterogeneous definitions or incomplete arm-level denominators, particularly anastomotic leak, were not pooled quantitatively and were instead summarized descriptively.

Pooled log odds ratios were estimated using an inverse-variance random-effects model with the DerSimonian–Laird estimator for between-study variance ($\tau^{2}$) [23,24]. A random-effects model was selected a priori because clinical and methodological heterogeneity was expected across trials, including differences in oral antibiotic regimens, timing of administration, patient populations, surgical procedures, and SSI definitions. Pooled effects were back-transformed and reported as odds ratios with 95% confidence intervals. In addition to the pooled odds ratio and 95% confidence interval, we calculated 95% prediction intervals for the main random-effects meta-analyses. Whereas the confidence interval quantifies the uncertainty around the average intervention effect, the prediction interval estimates the range of true effects that might be expected in a future comparable study or clinical setting. Prediction intervals were calculated on the log odds ratio scale as the pooled estimate plus or minus $t_{0.975,k-2}\sqrt{SE{\left(\hat{\mu }\right)}^{2}+\tau^{2}}$, where *k* is the number of studies included in the corresponding meta-analysis, and were then back-transformed to the odds ratio scale. Prediction intervals were interpreted cautiously when the number of studies was small because estimation of $\tau^{2}$ and the resulting interval may be imprecise [23]. A two-sided *p* value <0.05 was considered statistically significant.

Statistical heterogeneity was assessed using Cochran’s *Q* statistic, $\tau^{2}$, and $I^{2}$ [23,25]. $I^{2}$ was interpreted as the percentage of total variability in observed effect estimates attributable to between-study heterogeneity rather than sampling error. According to Cochrane guidance, $I^{2}$ values of approximately 0–40% may not be important, 30–60% may represent moderate heterogeneity, 50–90% may represent substantial heterogeneity, and 75–100% may represent considerable heterogeneity. These thresholds were considered approximate and were interpreted together with $\tau^{2}$, the direction and magnitude of effects, any available prediction intervals, and the clinical and methodological diversity across trials. $I^{2}$ values were interpreted cautiously, particularly in analyses including few studies, because uncertainty around heterogeneity estimates may be substantial.

The primary analysis evaluated overall SSI. Secondary analyses were performed for incisional SSI and organ-space SSI when arm-level data were available. Prespecified exploratory subgroup analyses were performed for trials using metronidazole-containing oral antibiotic regimens and according to type of surgery when sufficiently comparable data were reported. Leave-one-out sensitivity analysis for organ-space SSI was performed by sequentially excluding one study at a time and recalculating the pooled estimate. In addition, a sensitivity analysis excluding studies judged to be at high overall risk of bias was performed for the primary outcome.

Potential small-study effects and reporting bias were explored for the primary outcome using visual inspection of funnel plots and Egger’s regression test [23,26]. Because only 12 studies were included, these analyses were considered exploratory and were interpreted cautiously.

### 2.9. Heterogeneity and Subgroup Analyses

Subgroup analyses were prespecified for metronidazole-containing regimens and surgery type. These subgroup analyses were exploratory. Given the small number of non-metronidazole trials, no formal interaction test was performed to determine whether metronidazole modified the treatment effect. Additional subgroup analyses by operative approach, pathology, or anastomotic configuration were not feasible because these variables were inconsistently reported at the trial-arm level across the included studies.

### 2.10. Publication Bias

For clarity, publication-bias assessment in this review consisted of visual funnel-plot inspection and Egger’s regression test for the primary outcome; given the limited number of included trials, these analyses were considered exploratory rather than confirmatory [23,26].

### 2.11. Certainty of Evidence

Certainty of evidence for the primary outcome and key secondary outcomes was assessed using the GRADE approach [27], considering risk of bias, inconsistency, indirectness, imprecision, and publication bias. Judgments were made on an outcome-specific basis and incorporated the results of sensitivity analyses when available. Exploratory subgroup analyses were summarized separately and interpreted cautiously. The detailed GRADE assessment is provided in Supplementary Tables S5 and S6.

## 3. Results

### 3.1. Study Selection

The database search identified a total of 66 records, including 19 from PubMed, 33 from the Cochrane Library, and 14 from Scopus. After removal of 18 duplicates, 48 records underwent title and abstract screening. Of these, 23 full-text reports were assessed for eligibility. Consultation of ClinicalTrials.gov did not identify additional eligible reports.

After full-text review, 11 studies were excluded for predefined reasons, including failure to make the direct comparison of interest (MBP+OAB versus MBP alone), insufficient reporting of SSI outcomes, inclusion of pediatric populations, omission of standard intravenous prophylaxis, or alternative comparator groups. Ultimately, 12 randomized controlled trials fulfilled all inclusion criteria and were included in the quantitative synthesis. The study selection process is summarized in the PRISMA flow diagram (Figure 1).

### 3.2. Study Characteristics

The 12 included trials were published between 2005 and 2024 and comprised a total of 4073 patients, of whom 2069 received mechanical bowel preparation plus oral antibiotics and 2004 received mechanical bowel preparation alone. For Espín-Basany et al. (2005) [28], the two oral-antibiotic arms were combined into a single MBP+OAB group for pairwise comparison with the MBP-alone arm, so each randomized participant contributed only once to the meta-analysis. All studies were parallel-group randomized controlled trials. Four were multicentre studies, whereas eight were conducted at single centres. Sample sizes ranged from 91 to 579 patients per trial.

All trials evaluated adults undergoing elective colorectal procedures, although the surgical populations varied and included mixed colorectal surgery, rectal resection, Crohn’s disease surgery, ulcerative colitis with ileal pouch–anal anastomosis, colon cancer surgery, and laparoscopic colorectal resection. Standard intravenous antibiotic prophylaxis was administered in both study arms in all trials. In the intervention arms, oral antibiotics were administered preoperatively in addition to mechanical bowel preparation, whereas control groups received mechanical bowel preparation alone. Blinding also varied across studies, ranging from double-blind and assessor- or rater-blinded designs to open-label trials.

Oral antibiotic regimens varied across studies. Most trials used combinations of an aminoglycoside (neomycin or kanamycin) with metronidazole, whereas individual trials used metronidazole with levofloxacin, kanamycin alone, or kanamycin with erythromycin. Timing schedules ranged from two split doses on the day before surgery to once-daily administration for three consecutive preoperative days. Follow-up duration ranged from 7 days to 90 days in studies with clearly reported surveillance intervals; Rybakov et al. (2021) [29] used a 30-day surveillance window, whereas Horie (2007) [30] did not clearly report follow-up duration.

Core study characteristics are summarized in Table 1, whereas expanded study characteristics together with the study-level extracted numerical data are provided in Supplementary Table S3A–D.

Of the 12 trials, 10 included metronidazole as part of the oral antibiotic regimen. Overall SSI rates in the control groups ranged from approximately 6% to 28%, reflecting differences in patient populations and surgical complexity.

### 3.3. Risk-of-Bias Assessment

**Figure 2:**
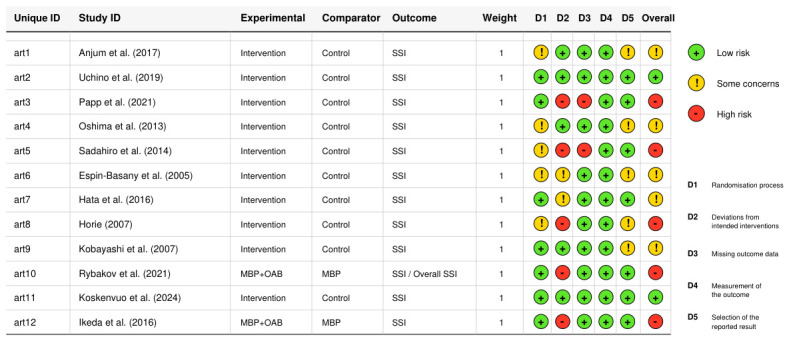
Risk-of-bias summary across included randomized controlled trials [28,29,30,31,32,33,34,35,36,37,38,39].

Two trials were judged to be at low overall risk of bias, five trials were rated as having some concerns, and five trials were judged to be at high overall risk of bias for the primary outcome. High-risk judgments were mainly driven by concerns in deviations from intended interventions and/or missing outcome data, whereas outcome measurement was generally judged to be at low risk. Because these methodological limitations could affect confidence in the pooled estimates, risk-of-bias judgments were incorporated into the GRADE assessment and into a sensitivity analysis excluding studies at high overall risk of bias. The risk-of-bias judgments across studies are summarized in Figure 2.

### 3.4. Primary Outcome: Overall Surgical Site Infection

Pooled analysis of all 12 randomized controlled trials demonstrated a significant reduction in postoperative surgical site infections with the use of oral antibiotics in addition to mechanical bowel preparation. The overall SSI incidence was 8.4% (174/2069) in the MBP+OAB group compared with 14.7% (295/2004) in the MBP-only group.

**Figure 3:**
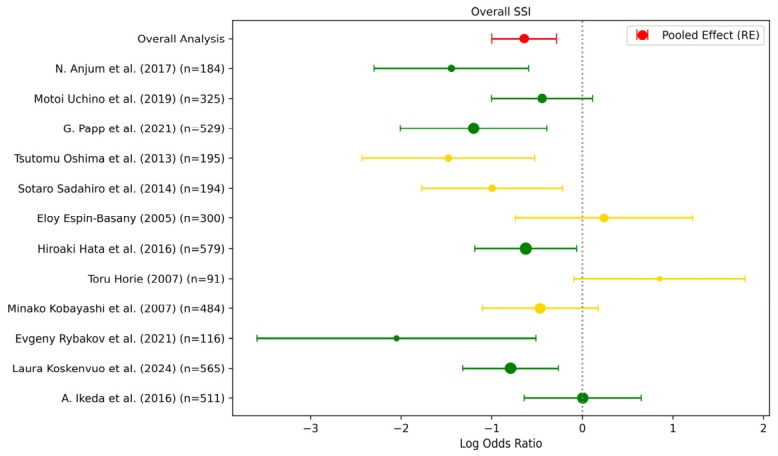
Forest plot of the primary outcome: overall surgical site infection (pooled OR 0.53, 95% CI 0.37–0.75; 95% prediction interval 0.17–1.66) [28,29,30,31,32,33,34,35,36,37,38,39].

The pooled odds ratio for overall SSI was 0.53 (95% CI 0.37–0.75; *p* < 0.001), indicating a statistically significant reduction in the odds of SSI with oral antibiotics. The corresponding 95% prediction interval was 0.17–1.66, indicating that although the average pooled effect favored MBP+OAB, a future comparable trial could plausibly show little or no clear benefit. A forest plot of the primary outcome is shown in Figure 3.

Most individual trials demonstrated effect estimates favoring the intervention, although the magnitude of benefit varied across studies.

### 3.5. Heterogeneity and Sensitivity Analyses

**Figure 4:**
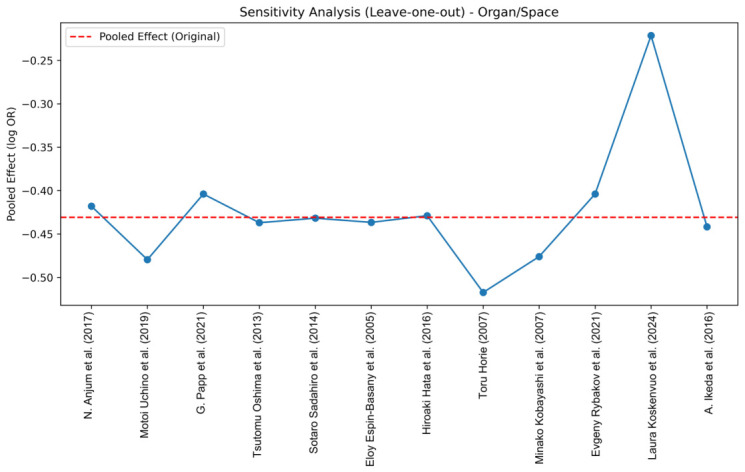
Leave-one-out sensitivity analysis for organ-space SSI [28,29,30,31,32,33,34,35,36,37,38,39].

For the primary outcome, heterogeneity was moderate-to-substantial (I^2^ = 62.5%, $\tau^{2}$ = 0.231), indicating relevant between-study variability in the magnitude of the treatment effect. However, most individual studies favored the MBP+OAB strategy, suggesting that heterogeneity mainly reflected differences in effect size rather than conflicting directions of effect. For organ-space SSI, leave-one-out sensitivity analysis suggested that the pooled estimate varied only modestly across iterations and was not dominated by any single study (Figure 4).

For the primary outcome, a sensitivity analysis excluding the five trials judged to be at high overall risk of bias showed a modest attenuation of the pooled effect without loss of statistical significance. The excluded studies were Papp et al. (2021) [33], Sadahiro et al. (2014) [35], Horie (2007) [30], Rybakov et al. (2021) [29], and Ikeda et al. (2016) [39]. This analysis yielded a pooled odds ratio of 0.50 (95% CI 0.36–0.71; *p* < 0.001), with reduced heterogeneity (I^2^ = 40.5%, $\tau^{2}$ = 0.081). The corresponding 95% prediction interval narrowed to 0.22–1.18, supporting the robustness of the direction of effect while still reflecting residual uncertainty around the null. These findings suggest that the observed reduction in overall SSI was not primarily driven by studies at high risk of bias. A detailed summary of the risk-of-bias sensitivity analysis is provided in Supplementary Table S4.

### 3.6. Secondary Outcomes

#### 3.6.1. Incisional Surgical Site Infection

**Figure 5:**
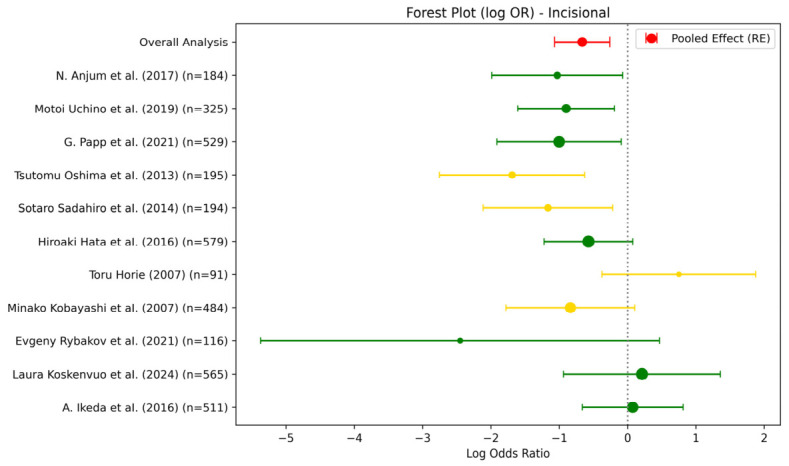
Forest plot for incisional surgical site infection (pooled OR 0.52, 95% CI 0.34–0.80; 95% prediction interval 0.14–1.96) [29,30,31,32,33,34,35,36,37,38,39].

Analysis of incisional SSI demonstrated a significant reduction with oral antibiotics. The pooled odds ratio was 0.52 (95% CI 0.34–0.80; *p* = 0.003), corresponding to an approximate 48% reduction in odds compared with MBP alone. Heterogeneity for this outcome was moderate (I^2^ = 57.5%, $\tau^{2}$ = 0.305), suggesting some between-study variability in the magnitude of effect. The corresponding 95% prediction interval was 0.14–1.96, indicating considerable predictive uncertainty despite the favorable average effect. The forest plot for this outcome is shown in Figure 5.

#### 3.6.2. Organ-Space Surgical Site Infection

**Figure 6:**
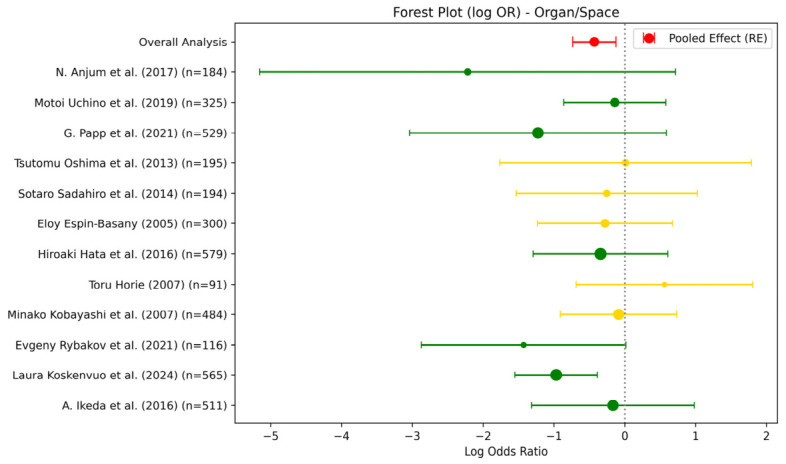
Forest plot for organ-space surgical site infection (pooled OR 0.63, 95% CI 0.45–0.88; 95% prediction interval 0.37–1.08) [28,29,30,31,32,33,34,35,36,37,38,39].

Oral antibiotic preparation was also associated with a significant reduction in organ-space SSIs. The pooled odds ratio was 0.63 (95% CI 0.45–0.88; *p* = 0.007). Heterogeneity for this outcome was low and likely not important (I^2^ = 8.3%, $\tau^{2}$ = 0.029), suggesting a more consistent effect across studies. The corresponding 95% prediction interval was 0.37–1.08, indicating a more consistent effect than for other outcomes but still allowing the null in a future comparable study. The corresponding forest plot is presented in Figure 6.

A separate sensitivity analysis excluding high-risk-of-bias studies was not performed for this secondary outcome because the number of available studies and events was limited.

#### 3.6.3. Exploratory Outcome: Anastomotic Leak

Anastomotic leak was considered a clinically relevant exploratory outcome. However, formal meta-analysis was not performed because leak definitions, denominators, and reporting formats were inconsistent across the included trials. In particular, some studies reported overall anastomotic leak, others used anastomotic dehiscence or suture failure, and not all provided arm-level denominators restricted to patients with an anastomosis.

Of the 12 included trials, 9 reported anastomotic leak, anastomotic dehiscence, or a closely related outcome as a distinct or semi-distinct endpoint, whereas 3 did not report it separately. Reporting varied substantially, with some studies embedding leakage within organ-space SSI and more recent rectal surgery trials applying graded definitions. Table 2 summarizes how these data were reported across the included studies.

Overall, the reported findings did not allow a definitive conclusion regarding the effect of oral antibiotic bowel preparation on anastomotic leak. The direction of effect was not consistently assessable across studies because event definitions, denominators, and reporting formats differed substantially. Nevertheless, this complication remains highly relevant given its close relationship with intra-abdominal infection and organ-space SSI.

### 3.7. Subgroup Analyses

#### 3.7.1. Metronidazole-Containing Regimens

**Figure 7:**
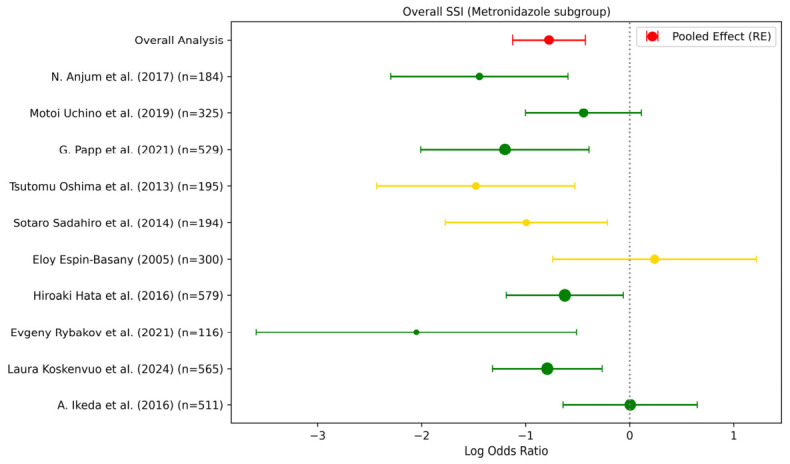
Forest plot for the metronidazole-containing subgroup analysis (pooled OR 0.46, 95% CI 0.33–0.65; 95% prediction interval 0.17–1.25). Study labels report the publication year and the sample size included in each trial. Effect estimates are displayed on the log odds ratio scale, with the vertical dashed line indicating the null effect [28,29,31,32,33,34,35,36,38,39].

In the subgroup of 10 trials in which the oral antibiotic regimen included metronidazole, the pooled odds ratio for overall SSI was 0.46 (95% CI 0.33–0.65; *p* < 0.001), indicating that the overall treatment effect was preserved in this subset. Heterogeneity remained moderate (I^2^ = 53.0%, $\tau^{2}$ = 0.156), likely reflecting differences in companion antibiotics, dosing schedules, study populations, and perioperative protocols. The corresponding 95% prediction interval was 0.17–1.25, indicating that effects in a future comparable trial could be smaller and potentially compatible with no clear benefit. Because most included trials used metronidazole-containing regimens and only a small number did not, this analysis should be interpreted as exploratory and hypothesis-generating rather than as evidence that metronidazole was the specific driver of benefit or superior to alternative oral antibiotic strategies. No formal interaction test was performed. The forest plot for this subgroup is shown in Figure 7.

#### 3.7.2. Type of Surgery

Two trials exclusively enrolling rectal surgery patients demonstrated a pooled odds ratio of 0.30 (95% CI 0.09–0.95; *p* = 0.04), suggesting a pronounced reduction in SSI risk with oral antibiotics. However, this finding is based on a limited number of studies and should be interpreted cautiously. In contrast, trials including mixed or non-rectal colorectal surgery populations showed a pooled odds ratio of 0.57 (95% CI 0.39–0.85; *p* = 0.006), with substantial heterogeneity (I^2^ = 64.3%, $\tau^{2}$ = 0.251). A summary of subgroup analyses is provided in Table 3.

### 3.8. Publication Bias Assessment

**Figure 8:**
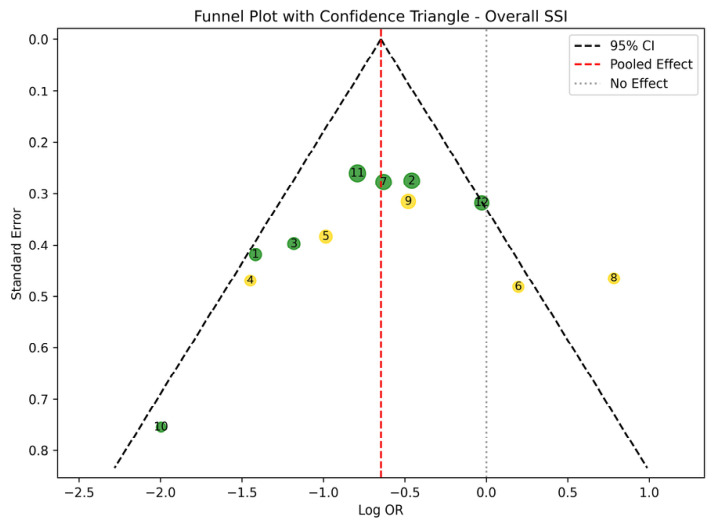
Funnel plot for the primary outcome (overall surgical site infection) used for exploratory assessment of reporting bias and small-study effects.

Visual inspection of the funnel plot for the primary outcome did not show marked asymmetry, and Egger’s regression test was not statistically significant (*p* = 0.37). However, because only 12 studies were included, the assessment of publication bias had limited power and should be interpreted cautiously. Therefore, the absence of statistically significant asymmetry should not be taken as evidence that publication bias or small-study effects are absent. The corresponding funnel plot is shown in Figure 8.

### 3.9. Certainty of Evidence

Certainty of evidence for the primary outcome and key secondary outcomes was assessed using the GRADE approach [27], considering risk of bias, inconsistency, indirectness, imprecision, and publication bias. Judgments were made on an outcome-specific basis and incorporated the results of sensitivity analyses when available. Overall SSI and organ-space SSI were rated as moderate-certainty evidence, incisional SSI as low-certainty evidence, and anastomotic leak as very low-certainty evidence. Exploratory subgroup analyses were summarized separately and interpreted cautiously. The detailed GRADE assessment is provided in Supplementary Tables S5 and S6.

## 4. Discussion

### 4.1. Comparison with Previous Evidence

Our results are aligned with prior pooled analyses that reported lower SSI rates when oral antibiotics were combined with mechanical preparation [10,11]. The overall effect size in the present study (OR around 0.5) is comparable to the 40–50% relative risk reduction reported in earlier meta-analyses. Notably, some previous studies identified a clearer signal for reduction in incisional SSI than for organ-space SSI. In contrast, our analysis demonstrated significant reductions in both outcomes, including organ-space infection (OR 0.63, 95% CI 0.45–0.88). A plausible explanation is the inclusion of more recent large RCTs, which increased power to detect effects on less frequent deep infections [38].

Our findings also corroborate the conclusions of recent high-level syntheses, including the latest Cochrane review and a network meta-analysis, both of which support combined oral antibiotic plus mechanical bowel preparation strategies [12,13]. In addition, our subgroup analyses help explain heterogeneity seen in earlier pooled work by showing more homogeneous effects in specific endpoints, especially organ-space infections.

### 4.2. Heterogeneity and Quality Considerations

Moderate overall heterogeneity (I^2^ around 60%) was expected given differences in antibiotic regimens, case mix, underlying pathology, rectal versus colonic surgery, anastomotic risk, study era, institutional protocols, perioperative pathways, and SSI definitions. Importantly, by restricting inclusion to trials in which both groups received standard intravenous prophylaxis, the incremental effect is attributable specifically to oral prophylaxis. This design choice strengthens internal validity relative to older pooled studies that mixed broader comparator strategies.

The subgroup analyses should be interpreted cautiously. Effects remained favorable in metronidazole-containing regimens, and heterogeneity was substantially lower for organ-space SSI, suggesting a stable benefit for deep infection prevention. However, because most included trials used metronidazole-containing regimens, the former observation reflects consistency of the overall effect within that subset rather than proof that metronidazole was the specific driver of benefit. We also observed a potentially larger relative effect in rectal surgery subsets, consistent with the higher baseline risk of pelvic sepsis in rectal procedures.

Although the pooled estimate remained robust in sensitivity analyses, it should be interpreted as an average effect across clinically heterogeneous elective colorectal surgery populations. Additional subgroup analyses by surgical approach, pathology, or anastomotic configuration were not feasible because these variables were inconsistently reported at arm level across the included trials, and post hoc stratification would have risked ecological bias.

The risk-of-bias assessment was also incorporated into the interpretation of the findings. Although several included trials had methodological limitations, the sensitivity analysis excluding studies at high overall risk of bias showed a consistent effect estimate for the primary outcome (OR 0.50, 95% CI 0.36–0.71; *p* < 0.001). Therefore, risk of bias was not considered sufficient to downgrade the certainty of evidence for overall SSI. However, the certainty of evidence was downgraded for inconsistency because heterogeneity remained relevant in the main analysis. For secondary outcomes, certainty was assessed individually. Incisional SSI was rated as low-certainty evidence because of risk-of-bias concerns and heterogeneity, whereas organ-space SSI was rated as moderate-certainty evidence because heterogeneity was low and the confidence interval excluded no effect. Evidence regarding anastomotic leak remained very uncertain because of inconsistent definitions and reporting across studies.

### 4.3. Clinical Implications

These findings have immediate practical implications. Adding oral antibiotics to standard preoperative preparation can substantially reduce SSI risk, potentially improving recovery, reducing length of stay, and lowering readmissions and costs. Importantly, benefit was observed on top of intravenous prophylaxis, indicating that oral prophylaxis provides additive protection rather than replacing standard perioperative antibiotics.

Typical oral regimens (for example, aminoglycoside plus metronidazole the day before surgery) are low-cost and generally well tolerated. Serious adverse events attributable to oral prophylaxis were not a dominant signal across included trials. Although clinicians should remain vigilant for antibiotic-related adverse effects and microbiome perturbation, the short preoperative course used in these protocols appears to have a favorable risk-benefit profile.

From an antimicrobial stewardship perspective, the use of oral antibiotics before elective colorectal surgery warrants careful consideration. Although oral antibiotic bowel preparation is delivered as a short, protocolized preoperative prophylactic intervention rather than as prolonged therapeutic antibiotic exposure, it nevertheless increases antimicrobial use. Therefore, its implementation should be aligned with antimicrobial stewardship principles, including appropriate patient selection, locally adapted regimens, consideration of institutional resistance patterns, and avoidance of unnecessary postoperative antibiotic continuation [40].

The potential benefits of oral antibiotic bowel preparation in reducing surgical site infection must be balanced against possible ecological harms, including selection of resistant organisms and disruption of the intestinal microbiome. Antibiotic exposure has been associated with increased antimicrobial resistance at the individual patient level, and microbiome studies suggest that antibiotic-related disruption of the gut microbiota may persist beyond the immediate treatment period [41,42]. Available colorectal surgery data do not show a consistent increase in postoperative *Clostridioides difficile* infection associated with preoperative oral antibiotics; however, this outcome has been inconsistently reported across studies [43]. Moreover, most trials were not designed to evaluate antimicrobial resistance, microbiome disruption, or long-term ecological safety. Future studies should prospectively assess stewardship-relevant endpoints, including *C. difficile* infection, microbiome recovery, colonization or infection by multidrug-resistant organisms, and the impact of locally adapted oral antibiotic protocols.

The present meta-analysis specifically evaluates the incremental effect of adding oral antibiotic bowel preparation to mechanical bowel preparation and should not be interpreted as a comparison of all available bowel preparation strategies. This distinction is clinically relevant because the contemporary debate has shifted from whether oral antibiotics improve outcomes when added to mechanical preparation, to whether oral antibiotics can be used without mechanical preparation and whether mechanical bowel preparation is necessary at all.

Available evidence suggests that oral antibiotics may provide benefit even in the absence of mechanical bowel preparation, particularly in colon surgery. In the ORALEV randomized trial, preoperative oral antibiotics without mechanical bowel preparation reduced surgical-site infections compared with no oral antibiotics in elective colon surgery. Conversely, the MOBILE trial did not demonstrate a reduction in surgical-site infections or overall morbidity with combined mechanical and oral antibiotic bowel preparation compared with no bowel preparation in elective colectomy. Network meta-analyses have suggested that strategies including oral antibiotics, with or without mechanical bowel preparation, may be among the most effective approaches, but the independent contribution of mechanical bowel preparation remains uncertain. Evidence may also differ between colon and rectal surgery; notably, the MOBILE2 trial reported benefit from adding oral antibiotics to mechanical bowel preparation in elective rectal resection [9,38,44,45].

Therefore, our findings support the benefit of adding oral antibiotics in settings where mechanical bowel preparation is used, but they do not establish that mechanical bowel preparation is necessary. Future trials should directly compare oral antibiotics alone versus mechanical plus oral antibiotic bowel preparation, ideally stratified by colon versus rectal surgery, minimally invasive versus open surgery, and anastomotic risk profile.

### 4.4. Metronidazole in Oral Prophylaxis

The subgroup findings show consistency of the overall effect among trials using metronidazole-containing regimens, but they do not establish that metronidazole is the specific driver of benefit. Because most included trials used metronidazole-containing oral regimens, this subgroup comparison was highly imbalanced and was not designed to determine whether metronidazole modifies the treatment effect or is superior to alternative oral antibiotic strategies. In addition, the oral regimens differed in other components, doses, timing, surgical populations, and perioperative protocols. Given the limited number of non-metronidazole trials and the absence of a sufficiently powered interaction analysis, this finding should be interpreted cautiously and considered hypothesis-generating. Anaerobic coverage remains biologically important in colorectal infection prevention, which helps explain why metronidazole-containing combinations are commonly used in practice. Among the randomized trials included in this review, metronidazole-containing regimens encompassed neomycin-plus-metronidazole, kanamycin-plus-metronidazole, and fluoroquinolone-plus-metronidazole combinations, and the pooled subgroup result was compatible with benefit; however, comparative superiority over other oral antibiotic strategies cannot be inferred from these subgroup data [28,31,32,33,34,35,36,39].

### 4.5. Anastomotic Leak as an Exploratory Outcome

Anastomotic leak is clinically important and biologically linked to postoperative infection, organ-space SSI, and anastomotic integrity. For this reason, a descriptive synthesis of leak reporting across the included randomized trials was undertaken. However, the available data were too heterogeneous for formal pooling. Definitions ranged from suture dehiscence to clinically or radiologically graded leak, several studies embedded leak within organ-space SSI, and not all reports restricted denominators to patients with an anastomosis. Overall, the available data did not allow a definitive conclusion regarding the effect of oral antibiotic bowel preparation on anastomotic leak, although this question remains highly relevant and warrants focused future investigation [14].

### 4.6. Limitations

This study has several limitations. First, despite strict eligibility criteria and exclusive inclusion of randomized controlled trials, relevant clinical and methodological heterogeneity remained across studies spanning nearly two decades. Trials differed in surgical population, indication for surgery, proportion of colon versus rectal resections, operative approach, perioperative pathways, oral antibiotic regimens, timing and number of doses, and SSI definitions or surveillance periods. Important effect modifiers such as benign versus malignant pathology and anastomotic configuration were also inconsistently reported and could not be analyzed reliably at the trial-arm level. Therefore, the pooled estimate should be interpreted as an average treatment effect across heterogeneous elective colorectal surgery populations rather than as evidence of a uniform effect across all surgical subgroups.

Second, risk of bias was not uniform across included trials. Several studies had RoB 2 concerns, particularly in deviations from intended interventions and/or missing outcome data, although outcome measurement was generally judged to be at low risk. Together with trial-level reporting limitations, variability in outcome definitions, and incomplete reporting of adverse events, these issues reduce confidence in some secondary and subgroup estimates, even though the primary finding remained stable after exclusion of studies at high overall risk of bias.

Third, the review protocol was prespecified but was not prospectively registered in PROSPERO or another public registry. Although eligibility criteria, outcomes, and statistical analyses were defined before data extraction, the lack of prospective registration may reduce transparency regarding protocol adherence and possible deviations.

Fourth, the search strategy involved language and publication-period restrictions. Restricting the review to English-language publications may have introduced language bias and may have excluded potentially relevant studies published in other languages. Although empirical evaluations of English-language restrictions in conventional medical systematic reviews have not consistently shown major changes in pooled treatment estimates, the impact of language restriction is difficult to predict for an individual review and should therefore be acknowledged as a limitation [46]. In addition, limiting eligibility to studies published from 2005 onward may have excluded older randomized trials. This threshold was defined a priori to enhance clinical applicability because older trials were conducted before the widespread adoption of enhanced recovery pathways, modern infection-prevention practices, and minimally invasive colorectal surgery [17,18]. Nevertheless, this time restriction reduces the comprehensiveness of the review and represents a potential source of selection bias. Beyond consultation of ClinicalTrials.gov as a trial registry, a broader grey literature search was not performed. This may have led to omission of unpublished or non-peer-reviewed randomized evidence, although such sources often lack sufficient detail on intervention fidelity, outcome definitions, risk-of-bias domains, and arm-level event counts for reliable synthesis.

Fifth, this review was restricted to randomized controlled trials and did not include observational studies. This approach was chosen to prioritize internal validity and to provide a focused estimate of the efficacy of MBP+OAB versus MBP alone. However, exclusion of observational studies may limit the assessment of real-world effectiveness, implementation across heterogeneous clinical settings, rare adverse events, and antimicrobial stewardship outcomes. Inclusion of non-randomized studies would have required a separate prespecified methodology, including extraction of adjusted effect estimates and risk-of-bias assessment with tools designed for non-randomized intervention studies, such as ROBINS-I [19,20]. Observational evidence should therefore be regarded as complementary rather than directly interchangeable with the randomized evidence synthesized in this meta-analysis.

Sixth, the comparator evaluated in this meta-analysis was mechanical bowel preparation plus oral antibiotics versus mechanical bowel preparation alone. Consequently, the present study cannot determine whether oral antibiotics alone are equivalent or superior to combined mechanical and oral antibiotic preparation, nor can it assess whether mechanical bowel preparation is necessary. The conclusions should therefore be restricted to the incremental benefit of adding oral antibiotics when mechanical bowel preparation is already part of the perioperative protocol.

Seventh, the number of included trials was limited for several secondary and subgroup analyses. The assessment of publication bias was therefore underpowered because only 12 studies were included, and meta-regression was not performed because the available number of trials was too small and relevant covariates were unevenly distributed or inconsistently reported at trial-arm level. Such analyses would therefore have been underpowered, unstable, and at risk of ecological bias. Subgroup analyses, especially those based on few studies (e.g., rectal surgery), should therefore be interpreted cautiously. In particular, the metronidazole-containing subgroup was highly imbalanced, with few non-metronidazole trials, and no formal interaction test was performed; this finding should be regarded as exploratory and hypothesis-generating. Certainty of evidence also varied across non-primary outcomes: organ-space SSI was rated as moderate-certainty, incisional SSI as low-certainty, anastomotic leak as very low-certainty, and subgroup analyses remained exploratory.

Eighth, the lack of a pooled estimate for anastomotic leak is a further relevant limitation of this review. Although anastomotic leak is clinically important and biologically linked to infection prevention, heterogeneity and incompleteness of reporting across trials precluded a robust quantitative synthesis. Other clinically relevant outcomes, including overall morbidity, readmission, length of stay, mortality, patient-reported tolerability, and adverse events, were also not uniformly reported and could not be synthesized robustly.

Finally, oral antibiotic regimens varied across studies in drug combinations, doses, timing, and duration. The included trials were primarily designed to assess postoperative infectious complications and did not consistently report antimicrobial stewardship outcomes. Therefore, this meta-analysis cannot identify a single optimal oral antibiotic regimen or adequately quantify the impact of oral antibiotic bowel preparation on antimicrobial resistance, intestinal microbiome disruption, multidrug-resistant organism colonization, or *Clostridioides difficile* infection.

Future research should prioritize large, contemporary, and ideally double-blind randomized trials comparing standardized oral prophylaxis protocols, including direct comparisons of OAB alone versus MBP+OAB, stratified where possible by colon versus rectal surgery, minimally invasive versus open surgery, and anastomotic risk profile. These studies should evaluate broader clinical outcomes, including anastomotic leak, overall morbidity, patient-reported tolerability, and stewardship-relevant endpoints such as antimicrobial resistance ecology, microbiome recovery, and *C. difficile* infection. Future updates of this review should also consider a dedicated grey-literature search if sufficiently detailed unpublished or non-peer-reviewed randomized data become available.

Protocol standardization is also needed. Considerable between-trial variation in drug selection, dose timing, and bowel preparation likely contributes to heterogeneity and complicates translation into routine care. Consensus regimens adapted to local availability and resistance patterns would improve comparability and implementation.

### 4.7. Strengths and Overall Interpretation

The main strengths of this meta-analysis are the exclusive focus on randomized evidence, strict comparator structure, inclusion of recent high-quality trials, and consistent benefit across primary, secondary, and sensitivity analyses. The absolute reduction in SSI risk is clinically meaningful given the downstream burden of postoperative infection.

In summary, this updated evidence base indicates that preoperative oral antibiotic bowel preparation probably reduces surgical site infections when added to mechanical preparation and standard intravenous prophylaxis in elective colorectal surgery. The primary finding remained stable after exclusion of studies at high overall risk of bias and corresponds to moderate-certainty evidence, although heterogeneity and trial-level methodological limitations warrant cautious interpretation.

## 5. Conclusions

In conclusion, MBP plus oral antibiotics was associated with a reduction in overall SSI compared with MBP alone. This finding was supported by sensitivity analysis excluding high-risk-of-bias studies and was rated as moderate-certainty evidence. However, heterogeneity and methodological limitations across included trials warrant cautious interpretation. Evidence for secondary outcomes, particularly anastomotic leak and exploratory subgroup analyses, remains less certain. These findings support the addition of oral antibiotics to mechanical bowel preparation in settings where mechanical bowel preparation is used; however, they do not address whether oral antibiotics alone may be sufficient or whether mechanical bowel preparation can be safely omitted. Accordingly, these conclusions are most applicable to contemporary elective colorectal surgery practice rather than to historical perioperative settings and should be interpreted as based on randomized evidence focused on efficacy rather than on a comprehensive evaluation of all real-world observational data. 

## Figures and Tables

**Table 1:** Characteristics of the 12 included randomized controlled trials (RCTs).

Authors (Year)	Design/Blinding	Population/Procedure	Participants (n)			Oral Regimen	Timing/Schedule	Follow-Up	Outcomes Reported
			Random- ized	MBP+ OAB	MBP				
Anjum et al. (2017) [31]	Single-centre, double-blind, prospective RCT	Elective colorectal surgery	184	91	93	Metronidazole 400 mg + levofloxacin 200 mg	Three doses, day before surgery	30–90 d	Overall SSI; incisional SSI; organ-space SSI
Uchino et al. (2019) [32]	Randomized, rater-blinded, single-centre trial	Crohn’s disease surgery	325	163	162	Kanamycin 500 mg + metronidazole 500 mg	Three doses, day before surgery	30 d	Overall SSI; incisional SSI; organ-space SSI
Papp et al. (2021) [33]	Multicentre, prospective, assessor-blinded RCT	Elective colorectal surgery	529	253	276	Neomycin 2 g + metronidazole 500 mg	Three doses, day before surgery	30 d	Overall SSI; incisional SSI; organ-space SSI
Oshima et al. (2013) [34]	Randomized, non-blinded, single-centre trial	Ulcerative colitis/IPAA	195	97	98	Kanamycin + metronidazole	Three doses, day before surgery	30–90 d	Overall SSI; incisional SSI; organ-space SSI
Sadahiro et al. (2014) [35]	Single-centre, double-blind RCT	Elective colon cancer surgery	194	99	95	Kanamycin 0.5 g + metronidazole 0.5 g	Three doses, day before surgery	4 wk	Overall SSI; incisional SSI; organ-space SSI
Espín-Basany et al. (2005) [28]	Prospective randomized trial	Elective colorectal surgery	300	200	100	Neomycin 1 g + metronidazole 1 g	During MBP; one or three doses by study arm	7, 14, and 30 d	Overall SSI; incisional SSI; organ-space SSI
Hata et al. (2016) [36]	Multicentre, open-label RCT	Elective laparoscopic colorectal surgery	579	289	290	Kanamycin 1 g + metronidazole 750 mg	13 and 9 h before surgery	30 d	Overall SSI; incisional SSI; organ-space SSI
Horie (2007) [30]	Prospective randomized trial	Colorectal cancer surgery	91	46	45	Kanamycin 1500 mg	Once daily for 3 preoperative days	Unclear	Overall SSI; incisional SSI; organ-space SSI
Kobayashi et al. (2007) [37]	Randomized, open-label, multicentre trial	Colorectal cancer surgery	484	242	242	Kanamycin 1 g + erythromycin 400 mg	Three doses, day before surgery	6 wk	Overall SSI; incisional SSI; organ-space SSI
Rybakov et al. (2021) [29]	Single-centre, open, parallel-group RCT	Rectal surgery	116	57	59	Erythromycin 500 mg + metronidazole 500 mg	Three doses after starting MBP	30 d	Overall SSI; incisional SSI; organ-space SSI
Koskenvuo et al. (2024) [38]	Multicentre, double-blind, placebo-controlled RCT	Rectal resection	565	277	288	Neomycin 1 g + metronidazole 1 g, or kanamycin 1 g + metronidazole 750 mg	Two doses, day before surgery	30 d	Overall SSI; incisional SSI; organ-space SSI
Ikeda et al. (2016) [39]	Prospective randomized trial	Laparoscopic colorectal resection	511	255	256	Kanamycin 1 g + metronidazole 750 mg	Two doses, day before surgery	30 d	Overall SSI; incisional SSI; organ-space SSI

MBP = mechanical bowel preparation; OAB = oral antibiotic bowel preparation; IPAA = ileal pouch–anal anastomosis; RCT = randomized controlled trial; SSI = surgical site infection. Espín-Basany et al. [28] randomized 300 patients to three arms. For this meta-analysis, the two oral-antibiotic arms were combined as MBP+OAB (n = 200) and compared with the no-oral-antibiotic arm (MBP, n = 100), so each randomized participant contributed only once to the pairwise comparison.

**Table 2:** Reporting of anastomotic leak or related dehiscence across included randomized controlled trials.

Study	Reported?	Definition/Reporting Note
Anjum et al. (2017) [31]	Yes	Organ-space SSI attributed to anastomotic leak; diagnosis based on extraluminal contrast leakage on imaging or leakage identified at reoperation.
Uchino et al. (2019) [32]	No	Not reported as a distinct outcome.
Papp et al. (2021) [33]	Yes	Any clinically or radiologically proven anastomotic suture dehiscence counted as anastomotic leak.
Oshima et al. (2013) [34]	No	Not reported as a separate anastomotic leak endpoint.
Sadahiro et al. (2014) [35]	Yes	Reported as suture failure/leakage; leakage with abscess formation was counted within organ-space SSI.
Espín-Basany et al. (2005) [28]	Yes	Reported as suture dehiscence; no formal leak definition provided.
Hata et al. (2016) [36]	Yes	Presented as anastomotic leakage within the organ-space SSI subgroup; no explicit formal definition reported.
Horie (2007) [30]	Yes	SSI included wound infection and anastomotic leakage; no detailed leak definition identified.
Kobayashi et al. (2007) [37]	No	Not reported as a distinct outcome.
Rybakov et al. (2021) [29]	Yes	Graded anastomotic leak definition (A–C) based on a defect of the colorectal anastomosis causing communication between the intra- and extraluminal compartments.
Koskenvuo et al. (2024) [38]	Yes	Anastomotic dehiscence graded A–C, including asymptomatic radiologic or endoscopic cases.
Ikeda et al. (2016) [39]	Yes	Leak analysis was restricted to patients with an anastomosis; no formal definition was reported in the extracted trial text.

Study characteristics are summarized in Table 1. A “Yes” designation includes trials reporting leak, dehiscence, suture failure, or leakage as a distinct or semi-distinct outcome.

**Table 3:** Meta-analysis outcomes and subgroup analyses of surgical site infections.

Outcome	Pooled OR (95% CI)	95% Prediction Interval	I^2^ (%)	*p*-Value	Interpretation
Overall SSI (primary outcome)	0.53 (0.37–0.75)	0.17–1.66	62.5	<0.001	Significant average reduction; prediction interval crosses 1.0
Sensitivity excluding high-risk-of-bias studies	0.50 (0.36–0.71)	0.22–1.18	40.5	<0.001	Primary finding remains favorable; prediction interval narrows but still crosses 1.0
Incisional SSI	0.52 (0.34–0.80)	0.14–1.96	57.5	0.003	Significant average reduction; wide prediction interval crosses 1.0
Organ-space SSI	0.63 (0.45–0.88)	0.37–1.08	8.3	0.007	Significant average reduction; prediction interval narrowly crosses 1.0
Subgroup: Metronidazole regimens	0.46 (0.33–0.65)	0.17–1.25	53.0	<0.001	Exploratory subgroup; prediction interval crosses 1.0
Subgroup: Rectal surgery patients	0.30 (0.09–0.95)	–	–	0.04	Two studies only; prediction interval not calculated
Subgroup: Mixed/non-rectal colorectal surgery	0.57 (0.39–0.85)	–	64.3	0.006	Significant reduction consistent with overall analysis

SSI = surgical site infection; OR = odds ratio; CI = confidence interval; MBP = mechanical bowel preparation; I^2^ = heterogeneity statistic; “–” = not applicable or not calculated from the available subgroup data.

## Data Availability

The data supporting this study were extracted from previously published randomized controlled trials cited in the References. The manuscript, figures, and separate Supplementary Materials document report the review documentation and summary materials corresponding to the present synthesis, including Supplementary Tables S1, S2, S3A–D and S4–S6.

## Supplementary Materials

The following supporting information can be downloaded at https://www.mdpi.com/article/10.3390/medicina62061161/s1. Table S1: PRISMA 2020 checklist; Table S2: Full search strategies; Table S3A–D: Expanded study characteristics and study-level extracted numerical data used in the synthesis; Table S4: Sensitivity analysis according to risk of bias; Table S5: GRADE assessment of certainty of evidence for MBP+OAB versus MBP; Table S6: GRADE justification and clinical interpretation.
