# Supplementary material for: Adding Preoperative Oral Antibiotics to Mechanical Bowel Preparation Reduces Surgical Site Infections in Elective Colorectal Surgery: A Meta-Analysis of Randomized Controlled Trials
Source: Medicina (Kaunas). 2026 Jun 15;62(6):1161. doi: 10.3390/medicina62061161 (PMC13304088; doi:10.3390/medicina62061161)
Supplement: Supplementary file 1 [file medicina-62-01161-s001.zip › medicina-4267626-Supplementary Material.pdf]

Supplementary Materials

Adding Preoperative Oral Antibiotics to Mechanical Bowel Preparation Reduces Surgical Site Infections in Elective Colorectal Surgery: A Meta-Analysis of Randomized Controlled Trials

Héctor Guadalajara et al.

PRISMA Reporting and Search Strategy

This supplementary document contains Supplementary Tables S1, S2, S3A–S3D, and S4–S6.

Supplementary Table S1. PRISMA 2020 checklist.

Section	Item	Description	Location in manuscript
Title	1	Identify the report as a systematic review and meta-analysis.	Title page (p. 1)
Abstract	2	Provide a structured abstract aligned with PRISMA 2020 for abstracts.	Abstract (p. 1)
Introduction	3	Describe the rationale for the review in the context of existing knowledge.	Introduction (pp. 2–3)
Introduction	4	State the objectives or review question, including the PICO framework.	Introduction; Study Design and Protocol (p. 3)
Methods	5	Specify the eligibility criteria for study inclusion and exclusion.	Methods: Eligibility Criteria (p. 4)
Methods	6	Specify all information sources, including databases and trial registries, and the date last searched.	Methods: Literature Search Strategy (pp. 3–4); Supplementary Table S2
Methods	7	Present the full search strategies for all databases and other sources.	Methods: Literature Search Strategy (p. 4); Supplementary Table S2
Methods	8	Describe the methods used to decide whether a study met the inclusion criteria.	Methods: Study Selection (pp. 4–5)
Methods	9	Describe the methods used to collect data from reports.	Methods: Data Extraction (p. 5)
Methods	10a	List and define all outcomes for which data were sought.	Methods: Outcomes (pp. 5–6)
Methods	10b	List and define other variables for which data were sought.	Methods: Data Extraction (p. 5)
Methods	11	Specify the methods used to assess risk of bias in the included studies.	Methods: Risk-of-Bias Assessment (p. 5)
Methods	12	Specify the effect measures used for each outcome.	Methods: Statistical Analysis (p. 6)
Methods	13	Describe the synthesis methods, including heterogeneity and sensitivity analyses.	Methods: Statistical Analysis (pp. 6–7); Heterogeneity and Subgroup Analyses (p. 7)
Methods	14	Describe any methods used to assess risk of bias due to missing results in a synthesis.	Methods: Publication Bias (p. 7); Results: Publication Bias Assessment (p. 15)
Methods	15	Describe methods used to assess certainty of evidence.	Methods: Certainty of Evidence (p. 7); Results: Certainty of Evidence (p. 16); Supplementary Tables S5–S6
Results	16	Describe the results of the search and selection process, ideally using a flow diagram.	Results: Study Selection (p. 8); Figure 1 (p. 5)
Results	17	Cite and summarize the characteristics of each included study.	Results: Study Characteristics (p. 8); Table 1 (pp. 8–9); Supplementary Table S3A
Results	18	Present assessments of risk of bias for each included study.	Results: Risk-of-Bias Assessment; Figure 2 (p. 10)
Results	19	Present results for individual studies for each outcome.	Results (pp. 10–14); Figures 3, 5, and 6 (pp. 11–13); Supplementary Tables S3B–S3D; Figure 7 (p. 14) for metronidazole subgroup
Results	20	Present results of all syntheses, including heterogeneity and sensitivity analyses where performed.	Results: Primary Outcome, Secondary Outcomes, Subgroup Analyses, and Heterogeneity and Sensitivity Analyses (pp. 10–15); Table 3 (p. 15); Figures 3–7 (pp. 11–14); Supplementary Table S4
Results	21	Present assessments of reporting bias for each synthesis assessed.	Results: Publication Bias Assessment (p. 15); Figure 8 (p. 15)
Results	22	Present assessments of certainty of evidence for each outcome.	Results: Certainty of Evidence (p. 16); Supplementary Tables S5–S6
Discussion	23	Provide an interpretation of the results, limitations of the evidence, limitations of the review processes, and implications for practice and future research.	Discussion (pp. 16–21)
Other	24	Provide registration and protocol information, including registration status.	Methods: Study Design and Protocol (p. 3)
Other	25	Describe sources of financial or non-financial support.	Funding (p. 21)
Other	26	Declare competing interests.	Conflicts of Interest (p. 21)
Other	27	Report the availability of data, analytic materials, and supplementary information.	Supplementary Materials (p. 21); Data Availability (p. 21)

Supplementary Table S2. Full search strategies.

Source	Search strategy	Limits / notes	Records retrieved
PubMed	`("Bowel Preparation"[Mesh] OR "Mechanical Bowel Preparation" OR "Oral Antibiotics" OR "Enema") AND ("Colorectal Surgery"[Mesh] OR "Colectomy"[Mesh] OR "Colon Surgery" OR "Rectal Surgery") AND ("Antibiotic Prophylaxis"[Mesh] OR "Intravenous Antibiotics" OR "Oral Antibiotics") AND ("Randomized Controlled Trial"[Publication Type] OR randomized OR randomised OR trial)`	English language; January 2005–January 2025	19
Cochrane Library	`(("Randomized Controlled Trial" OR "RCT" OR randomized)) AND (("Bowel Preparation" OR "Mechanical Bowel Preparation" OR "Oral Antibiotics") AND ("Colorectal Surgery" OR "Colectomy" OR "Rectal Resection") AND ("Antibiotic Prophylaxis" OR "Preoperative Antibiotics")) NOT ("Pilot" OR "Feasibility" OR "Protocol")`	English language; January 2005–January 2025	33
Scopus	`("Randomized Clinical trial" AND "Bowel preparation") AND "Antibiotic" AND ("Colorectal surgery" OR "Colectomy" OR "Colon surgery") AND ("Postoperative complications" OR ("surgical" AND "site" AND "infection"))`	English language; January 2005–January 2025	14
ClinicalTrials.gov	Registry consultation using the core concepts colorectal surgery, bowel preparation, oral antibiotics, and randomized trials to identify potentially relevant registered or completed studies.	Consulted as a trial registry; no additional eligible reports were identified.	0

Expanded Study Characteristics and Extraction Data

Supplementary Tables S3A–S3D provide expanded study characteristics and the reconstructed outcome-specific 2 × 2 data used in the synthesis. Supplementary Table S3A summarizes expanded study characteristics and study-level arm sizes. Supplementary Tables S3B–S3D provide the reconstructed data for overall SSI, incisional SSI, and organ-space SSI, respectively. For Espín-Basany et al. (2005), the two oral-antibiotic arms were combined as a single MBP+OAB group (n = 200) and compared with the MBP group (n = 100), so all 300 randomized patients contributed once to the pairwise comparison.

Supplementary Table S3A. Expanded study characteristics and study-level arm sizes used in the synthesis.

Study	Design / blinding	Population / procedure	Rand.	Incl.	OAB	MBP	Oral regimen	Timing / schedule	Follow-up	Pooled outcomes
Anjum et al. (2017)	Single-centre, double-blind, prospective RCT	Elective colorectal surgery	184	184	91	93	Metronidazole 400 mg + levofloxacin 200 mg	Three doses, day before surgery	30–90 d	Overall SSI; incisional SSI; organ-space SSI
Uchino et al. (2019)	Randomized, rater-blinded, single-centre trial	Crohn's disease surgery	325	325	163	162	Kanamycin 500 mg + metronidazole 500 mg	Three doses, day before surgery	30 d	Overall SSI; incisional SSI; organ-space SSI
Papp et al. (2021)	Multicentre, prospective, assessor-blinded RCT	Elective colorectal surgery	529	529	253	276	Neomycin 2 g + metronidazole 500 mg	Three doses, day before surgery	30 d	Overall SSI; incisional SSI; organ-space SSI
Oshima et al. (2013)	Randomized, non-blinded, single-centre trial	Ulcerative colitis / IPAA	195	195	97	98	Kanamycin + metronidazole	Three doses, day before surgery	30–90 d	Overall SSI; incisional SSI; organ-space SSI
Sadahiro et al. (2014)	Single-centre, double-blind RCT	Elective colon cancer surgery	194	194	99	95	Kanamycin 0.5 g + metronidazole 0.5 g	Three doses, day before surgery	4 wk	Overall SSI; incisional SSI; organ-space SSI
Espín-Basany et al. (2005)	Prospective randomized trial	Elective colorectal surgery	300	300	200	100	Neomycin 1 g + metronidazole 1 g	During MBP; one or three doses by study arm	7, 14, and 30 d	Overall SSI; incisional SSI; organ-space SSI
Hata et al. (2016)	Multicentre, open-label RCT	Elective laparoscopic colorectal surgery	579	579	289	290	Kanamycin 1 g + metronidazole 750 mg	13 and 9 h before surgery	30 d	Overall SSI; incisional SSI; organ-space SSI
Horie (2007)	Prospective randomized trial	Colorectal cancer surgery	91	91	46	45	Kanamycin 1500 mg	Once daily for 3 preoperative days	Not clearly reported	Overall SSI; incisional SSI; organ-space SSI
Kobayashi et al. (2007)	Randomized, open-label, multicentre trial	Colorectal cancer surgery	484	484	242	242	Kanamycin 1 g + erythromycin 400 mg	Three doses, day before surgery	6 wk	Overall SSI; incisional SSI; organ-space SSI
Rybakov et al. (2021)	Single-centre, open, parallel-group RCT	Rectal surgery	116	116	57	59	Erythromycin 500 mg + metronidazole 500 mg	Three doses after starting MBP	30 d	Overall SSI; incisional SSI; organ-space SSI
Koskenvuo et al. (2024)	Multicentre, double-blind, placebo-controlled RCT	Rectal resection	565	565	277	288	Neomycin 1 g + metronidazole 1 g, or kanamycin 1 g + metronidazole 750 mg	Two doses, day before surgery	30 d	Overall SSI; incisional SSI; organ-space SSI
Ikeda et al. (2016)	Prospective randomized trial	Laparoscopic colorectal resection	511	511	255	256	Kanamycin 1 g + metronidazole 750 mg	Two doses, day before surgery	30 d	Overall SSI; incisional SSI; organ-space SSI

Supplementary Table S3B. Reconstructed 2×2 data for overall surgical site infection.

**Study**	**Included**	**MBP+ OAB events**	**MBP+ OAB non-events**	**MBP+ OAB total**	**MBP events**	**MBP non-events**	**MBP total**	**Continuity correction**
Anjum et al. (2017)	Yes	8	83	91	26	67	93	No
Uchino et al. (2019)	Yes	26	137	163	37	125	162	No
Papp et al. (2021)	Yes	8	245	253	27	249	276	No
Oshima et al. (2013)	Yes	6	91	97	22	76	98	No
Sadahiro et al. (2014)	Yes	11	88	99	24	71	95	No
Espín-Basany et al. (2005)	Yes	15	185	200	6	94	100	No
Hata et al. (2016)	Yes	21	268	289	37	253	290	No
Horie (2007)	Yes	17	29	46	9	36	45	No
Kobayashi et al. (2007)	Yes	17	225	242	26	216	242	No
Rybakov et al. (2021)	Yes	2	55	57	13	46	59	No
Koskenvuo et al. (2024)	Yes	23	254	277	48	240	288	No
Ikeda et al. (2016)	Yes	20	235	255	20	236	256	No
**Total**	–	**174**	**1,895**	**2,069**	**295**	**1,709**	**2,004**	–

Supplementary Table S3C. Reconstructed 2 × 2 data for incisional surgical site infection.

**Study**	**In-cluded**	**MBP+ OAB events**	**MBP+ OAB non-events**	**MBP+ OAB total**	**MBP events**	**MBP non-events**	**MBP total**	**Continuity correction**
Anjum et al. (2017)	Yes	7	84	91	23	70	93	No
Uchino et al. (2019)	Yes	12	151	163	27	135	162	No
Papp et al. (2021)	Yes	7	246	253	22	254	276	No
Oshima et al. (2013)	Yes	4	93	97	20	78	98	No
Sadahiro et al. (2014)	Yes	6	93	99	17	78	95	No
Espín-Basany et al. (2005)	Yes	15	185	200	6	94	100	No
Hata et al. (2016)	Yes	16	273	289	27	263	290	No
Horie (2007)	Yes	10	36	46	5	40	45	No
Kobayashi et al. (2007)	Yes	6	236	242	14	228	242	No
Rybakov et al. (2021)	Yes	0	57	57	5	54	59	Yes, 0.5
Koskenvuo et al. (2024)	Yes	6	271	277	5	283	288	No
Ikeda et al. (2016)	Yes	15	240	255	14	242	256	No
**Total**	–	**104**	**1,965**	**2,069**	**185**	**1,819**	**2,004**	–

Supplementary Table S3D. Reconstructed 2×2 data for organ-space surgical site infection.

**Study**	**In-cluded**	**MBP+ OAB events**	**MBP+ OAB non-events**	**MBP+ OAB total**	**MBP events**	**MBP non-events**	**MBP total**	**Continuity correction**
Anjum et al. (2017)	Yes	0	91	91	4	89	93	Yes, 0.5
Uchino et al. (2019)	Yes	15	148	163	17	145	162	No
Papp et al. (2021)	Yes	1	252	253	5	271	276	No
Oshima et al. (2013)	Yes	2	95	97	2	96	98	No
Sadahiro et al. (2014)	Yes	4	95	99	5	90	95	No
Espín-Basany et al. (2005)	Yes	4	196	200	3	97	100	No
Hata et al. (2016)	Yes	7	282	289	10	280	290	No
Horie (2007)	Yes	7	39	46	4	41	45	No
Kobayashi et al. (2007)	Yes	11	231	242	12	230	242	No
Rybakov et al. (2021)	Yes	2	55	57	9	50	59	No
Koskenvuo et al. (2024)	Yes	17	260	277	43	245	288	No
Ikeda et al. (2016)	Yes	5	250	255	6	250	256	No
**Total**	–	**75**	**1,994**	**2,069**	**120**	**1,884**	**2,004**	–

Note. For multi-arm trials, oral-antibiotic arms were combined into a single MBP+OAB group before effect estimation. Non-events were calculated as total minus events. A continuity correction of 0.5 was applied to all four cells only when at least one cell contained zero events. For incisional SSI, the endpoint was harmonized as total incisional SSI; when superficial and deep incisional SSI were reported separately, both were summed. For Espín-Basany et al., wound infection was used as the incisional SSI category, and suture dehiscence was used for the organ-space/anastomotic infectious complication category.

Risk-of-Bias Sensitivity and Certainty-of-Evidence Assessment

Supplementary Tables S4–S6 summarize the risk-of-bias sensitivity analysis and the GRADE-based certainty-of-evidence assessment for the present synthesis.

Supplementary Table S4. Sensitivity analysis according to risk of bias.

**Outcome**	**Main analysis**	**Sensitivity analysis excluding high-risk-of-bias studies**	**Interpretation**
Overall surgical site infection	174/2,069 vs 295/2,004; OR 0.53 (95% CI 0.37–0.75); $p < 0.001$; $I^2 = 62.5\%$; $\tau^2 = 0.231$; 95% PI 0.17–1.66	116/1,359 vs 202/1,273; OR 0.50 (95% CI 0.36–0.71); $p < 0.001$; $I^2 = 40.5\%$; $\tau^2 = 0.081$; 95% PI 0.22–1.18	The direction and magnitude of effect remained consistent after exclusion of the five high-risk studies.
Incisional SSI	104/2,069 vs 185/2,004; OR 0.52 (95% CI 0.34–0.80); $p = 0.003$; $I^2 = 57.5\%$; $\tau^2 = 0.305$; 95% PI 0.14–1.96	Not performed / not sufficiently informative because of the limited number of studies after exclusion	The result favors MBP+OAB, but certainty is limited by heterogeneity and methodological concerns.
Organ-space SSI	75/2,069 vs 120/2,004; OR 0.63 (95% CI 0.45–0.88); $p = 0.007$; $I^2 = 8.3\%$; $\tau^2 = 0.029$; 95% PI 0.37–1.08	Not performed / not sufficiently informative because of the limited number of events and studies	The result favors MBP+OAB, with low heterogeneity, but certainty remains affected by study-level limitations.
Anastomotic leak	Not pooled because of inconsistent reporting and definitions	Not applicable	The effect remains uncertain and was assessed narratively.
Overall SSI in metronidazole-containing regimens	Exploratory subgroup analysis favoring MBP+OAB; OR 0.46 (95% CI 0.33–0.65); $p < 0.001$; $I^2 = 53.0\%$; $\tau^2 = 0.156$; 95% PI 0.17–1.25	Not performed / not sufficiently informative as subgroup-level sensitivity analysis	Findings suggest preserved benefit within this subgroup but should be interpreted cautiously because this is an exploratory subgroup analysis.
Overall SSI in rectal surgery	Exploratory subgroup analysis based on two studies; OR 0.30 (95% CI 0.09–0.95); $p = 0.04$	Not applicable	Evidence is limited and imprecise; findings should not be overinterpreted.

Note. High-risk-of-bias studies excluded from the sensitivity analysis were Papp et al. (2021), Sadahiro et al. (2014), Horie (2007), Rybakov et al. (2021), and Ikeda et al. (2016).

Supplementary Table S5. GRADE assessment of certainty of evidence for MBP+OAB versus MBP.

**Outcome**	**Risk of bias**	**Inconsistency**	**Indirectness**	**Imprecision**	**Publication bias**	**Certainty of evidence**
Overall SSI	Not serious	Serious	Not serious	Not serious	Undetected, but assessment underpowered	Moderate
Incisional SSI	Serious	Serious	Not serious	Not serious	Not specifically evaluable	Low
Organ-space SSI	Serious	Not serious	Not serious	Not serious	Not specifically evaluable	Moderate
Anastomotic leak	Serious	Serious	Serious	Serious	Not evaluable	Very low

Supplementary Table S6. GRADE justification and clinical interpretation.

**Outcome**	**Justification**	**Clinical interpretation**
Overall SSI	Although some trials were judged to be at high risk of bias or to raise some concerns, the sensitivity analysis excluding high-risk-of-bias studies showed a consistent direction and magnitude of effect: OR 0.50 (95% CI 0.36–0.71; $p < 0.001$), with reduced heterogeneity ($I^2 = 40.5\%$; $\tau^2 = 0.081$). Therefore, risk of bias was not considered sufficient to downgrade this outcome. Certainty was downgraded for inconsistency because heterogeneity was substantial in the main analysis ($I^2 = 62.5\%$; $\tau^2 = 0.231$).	MBP+OAB probably reduces overall SSI compared with MBP alone, although heterogeneity limits certainty.
Incisional SSI	The effect estimate favored MBP+OAB, OR 0.52 (95% CI 0.34–0.80; $p = 0.003$), but heterogeneity was moderate ($I^2 = 57.5\%$; $\tau^2 = 0.305$), and the 95% prediction interval (0.14–1.96) crossed the null. This outcome may also be more vulnerable to differences in postoperative surveillance, diagnostic criteria, and outcome adjudication.	MBP+OAB may reduce incisional SSI, but certainty is limited.
Organ-space SSI	Some risk-of-bias concerns persisted at study level; however, inconsistency was low by point estimate ($I^2 = 8.3\%$; $\tau^2 = 0.029$), the 95% CI excluded no effect, OR 0.63 (95% CI 0.45–0.88; $p = 0.007$), and the 95% prediction interval was 0.37–1.08.	MBP+OAB probably reduces organ-space SSI, although the effect is more modest than for overall SSI.
Anastomotic leak	This outcome was not pooled because reporting and definitions were inconsistent across studies. The limited number of events and variability in definitions substantially reduced certainty.	The effect of MBP+OAB on anastomotic leak remains uncertain.
